# Identification of genome edited cells using CRISPRnano

**DOI:** 10.1093/nar/gkac440

**Published:** 2022-05-30

**Authors:** Thach Nguyen, Haribaskar Ramachandran, Soraia Martins, Jean Krutmann, Andrea Rossi

**Affiliations:** IUF – Leibniz Research Institute for Environmental Medicine, Duesseldorf, Germany; IUF – Leibniz Research Institute for Environmental Medicine, Duesseldorf, Germany; IUF – Leibniz Research Institute for Environmental Medicine, Duesseldorf, Germany; IUF – Leibniz Research Institute for Environmental Medicine, Duesseldorf, Germany; IUF – Leibniz Research Institute for Environmental Medicine, Duesseldorf, Germany

## Abstract

Genome engineering-induced cleavage sites can be resolved by non-homologous end joining (NHEJ) or homology-directed repair (HDR). Identifying genetically modified clones at the target locus remains an intensive and laborious task. Different workflows and software that rely on deep sequencing data have been developed to detect and quantify targeted mutagenesis. Nevertheless, these pipelines require high-quality reads generated by Next Generation Sequencing (NGS) platforms. Here, we have developed a robust, versatile, and easy-to-use computational webserver named CRISPRnano (www.CRISPRnano.de) that enables the analysis of low-quality reads generated by affordable and portable sequencers including Oxford Nanopore Technologies (ONT) devices. CRISPRnano allows fast and accurate identification, quantification, and visualization of genetically modified cell lines, it is compatible with NGS and ONT sequencing reads, and it can be used without an internet connection.

## Graphical Abstract

**Graphical Abstract:**
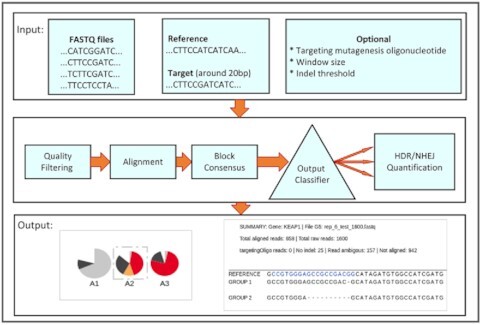
From input to output: fast and accurate identification, quantification and visualization of genome editing events using CRISPRnano.

## INTRODUCTION

Genome engineering technologies, including TALEN and CRISPR/Cas9 nuclease systems, have provided the ability to investigate the biological functions of genomic sequences (1,2). DNA double-strand breaks (DSBs) induced by genome editing technologies are mainly repaired by two mechanisms: non-homologous end joining (NHEJ) and homology-directed repair (HDR). DSB induced by the NHEJ repair pathway leads to the formation of indels (3,4) that can result in frameshift mutations and often to loss-of-function phenotypes. Its counterpart, the HDR pathway, can be exploited to introduce the desired mutation, useful to model human disease. Single cell cloning and subsequent sequencing are required to obtain cells that carry the desired genotype. T7 endonuclease assay, PCR or high-resolution melt analysis (HRMA) followed by Sanger sequencing are usually used to identify genetically modified alleles. Nevertheless, these experimental workflows are time-consuming, expensive, and impractical for large-scale screenings.

Deep sequencing at low sequencing output has been used to identify genetically modified cell clones using different software. Some of these software work as web-based applications that allow a fast and robust analysis of deep sequencing data to identify knockout and knock-in cells (5,6). However, the initial alignment algorithm of these software usually work only for gap-free sequences, which prevents the analysis of Oxford Nanopore Technologies (ONT) noisy data. Alternative software (6) integrate Needlemann Wunsch global alignment (7) that however performs poorly for sequences with arbitrary read length and different sequence boundaries. Other ‘non dedicated’ pipelines including Minimap2, Samtools, BCFtools (8,9) are robust for handling noisy data but are not user-friendly and run-on command lines. Thus, users are forced to follow a bioinformatic workflow that relies on run-on command lines and requires installing several third-party software under a Unix-like environment. In order to process non-uniform, noisy but inexpensive sequencing data, we developed a software named CRISPRnano that overcomes these issues and takes advantage of ONT platforms mobility. CRISPRnano allows a fast and accurate identification, quantification, and visualization of genetically modified bulk cell populations or individual clones and works as a webserver on the client-side. Moreover, CRISPRnano can handle NGS (e.g. Illumina and PacBio) and ONT data, does not require installation of third-party software, and takes full advantage of Nanopore sequencers’ portability by not requiring an internet connection.

## MATERIALS AND METHODS

### Maintenance of cells, CRISPR and transfection

HaCaT cells were maintained in Dulbecco's modified Eagle's medium (DMEM) (Gibco) supplemented with 10% of fetal bovine serum (FBS) (Gibco) and 1% of penicillin and streptomycin. Guide RNAs (gRNAs) were selected using (https://www.vbc-score.org/) (10), cloned into a pSpCas9(BB)-2A-GFP plasmid (AddGene #48138) and transfected into HEK 293T cells. gRNA efficiency was assessed by HRMA (3) using a MyGo PRO real-time PCR cycler (IT-IS Life Science LTD). High efficient gRNAs were used to transfect HaCAT cells using a Neon Electroporation system (Thermo). After 48hrs of transfection, GFP positive cells were FACS sorted. Bulk sorted cells were allowed to grow for some time and splitted into 96-well plates as single cells using limited dilution. 96-well plates with single cell clones were duplicated for maintenance and lysis (genotyping). Genomic DNA was isolated from the lysis plate using Proteinase K, PCR and analysis were performed as shown in Figure 2A and as described below. For CRISPR-Cas12a (Cpf1) based knock-in, gRNA, Cas12 protein and donor oligo were purchased from IDT and electroporated as ribonucleoprotein complexes (RNP) according to the manufacturer's protocol. After electroporation, cells were processed without FACS sorting. See supplementary information for the list of primers.

### DNA amplification and barcoding

DNA amplification was performed as previously described (4). Briefly, first-level PCR reactions were performed using 1 μl of lysate as a template in a 6.25 μl Phanta (Vazyme) PCR reaction according to the manufacturer's protocol (annealing temperature: 60°C; elongation time: 15 s, 19 cycles). PCR custom primers were designed to have adaptors for the second PCR. From this reaction, 2 μl was transferred to a second-level PCR using the same cycling conditions and a barcoded primer that is unique for each clone. Please note that Illumina and Nanopore barcodes are different.

### Library preparation and deep sequencing

PCR products were pooled, size-separated using a 1% agarose gel and purified using GeneJET gel extraction kit (Thermo Fisher Scientific). Illumina library preparation was performed as previously described (4). Illumina libraries were sequenced using an Illumina MiSeq benchtop sequencer, a Nano V2 reagent kit (2 × 150) in a single-end sequencing setup (251 cycles). ONT sequencing library was prepared using the Ligation Sequencing Kit (SQL-LSK109) according to the manufacturer's protocol and sequenced using a MinION MK1C or MinION using a Flongle flow cell and adapter. Data were obtained in FASTQ format and analyzed with CRISPRnano, Outknocker and CRISPResso2 (5,6) for comparison (supplementary information). CRISPRnano and Outknocker performance using bulk cells can be found in the supplementary information. ONT original data were processed by Guppy on Minion device, MinKNOW 21.05.25, MinKNOW Core 4.3.12, Bream 6.2.6, Guppy 5.0.16. The same data were re-run with a newer software version (Version 6.1.2 + e0556ff93) on the server with a SUP model to increase the quality score. The command used is: guppy_basecaller –min_qscore 7 –trim_barcodes –barcode_kits ‘SQK-LSK109’ -i all_fast5/ -s fastq/ -c dna_r9.4.1_450bps_sup.cfg -x auto. The Phred quality score can be increased from 19 to 21 (*Q*_median ∼ 21).

### Data input and adaptive alignment algorithm

As input, CRISPRnano takes multiple FASTQ files, up to 96 samples per run, that are the output of basecallers such as ont-guppy, bonito or Illumina. Briefly, reads with a score below the predefined quality threshold (*Q* = 7) are filtered out. The indel threshold determines at what relative occurrence an individual indel is considered unique (subsequences, Key = aligned_read[sgRNA_index – alpha: sgRNA_index + alpha] where alpha is the region of interest (default 20) defined in the input web form) of the respective clone analyzed, and is thus displayed in the alignment list. Low threshold values can result in false positive allele calling (e.g. sequencing errors), whereas high threshold values may result in false negative allele calling. Values in between 0 and 100% can be entered and the default value is 5%. FASTQ reads are aligned with the reference sequence segment based on the guide RNA binding sequence and PAM motif where the nuclease (e.g. Cas9) is predicted to induce the DSB. Importantly, the genomic window size can be adjusted around the predicted DSB, this step allows to remove unwanted indels or mutations that are outside the genomic region of interest. The software uses a two-step alignment to speed up the processing time. CRISPRnano controls whether the ONT or Illumina reads contain the genomic sequence that matches the reference genomic sequence thus ensuring that the proper read is selected and that other sequences are skipped. Smith Watermann local alignment algorithm (11) is embedded in the CRISPRnano code and uses a dynamic programming algorithm with an affine gap score matrix (12). Smith Watermann affine score alignment strategy can be found in the supplementary information. The generation of ground truth data test script, the comparison between CRISPRnano and other software and the CRISPR analysis workflow using Minimap2 and Samtools can be found in the supplementary information.

### Block consensus and webserver

Block consensus assessment is used to process the sequence alignment output. Thus, single base consensus is extended to block consensus. All similar sequences of a particular indel are stored and only the majority of the sequences greater than a predefined threshold are kept.

**Figure 1.**
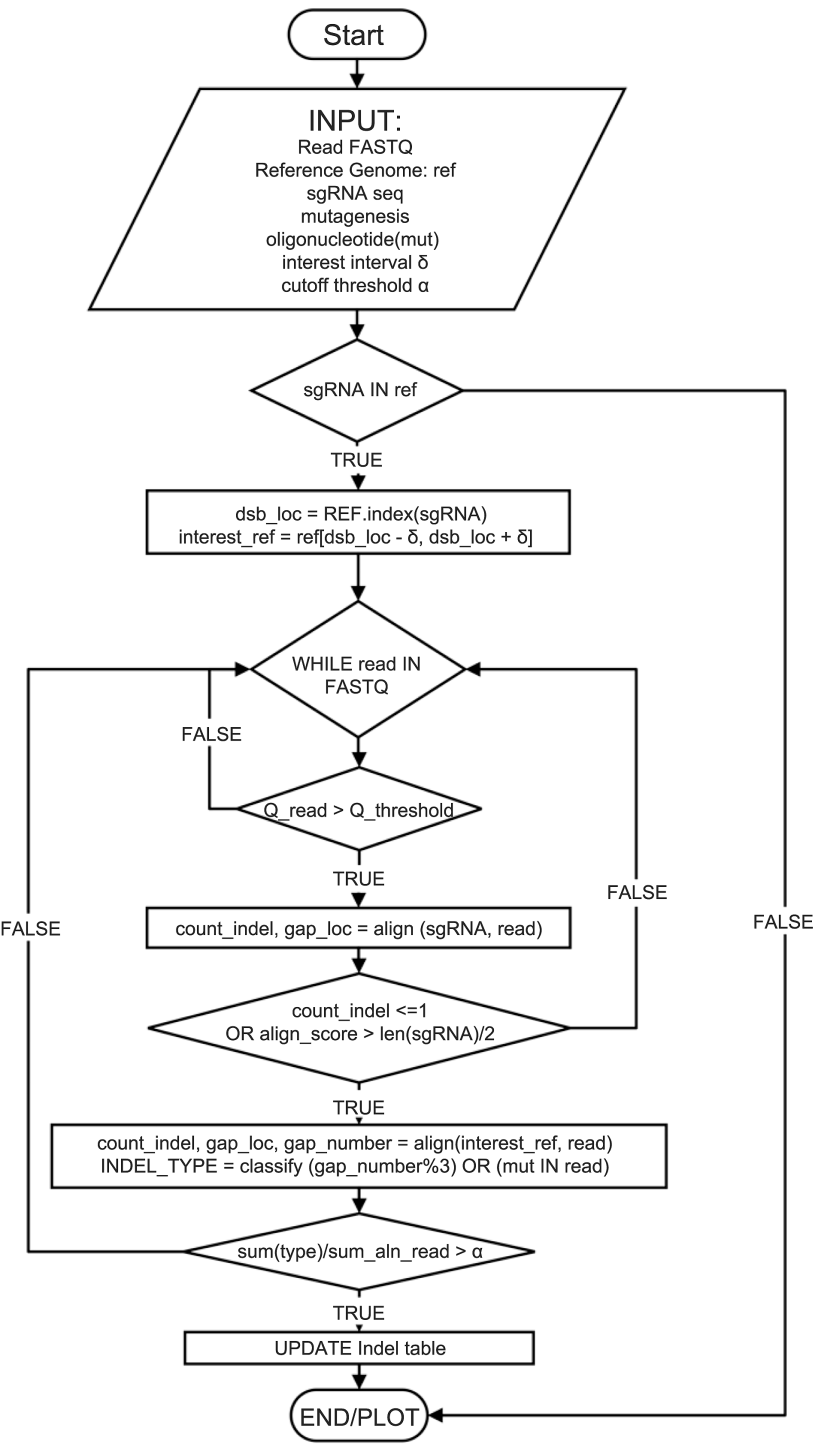
CRISPRnano workflow. A simplified flowchart describing CRISPRnano workflow from the input to the output is depicted. Up to 96 FASTQ files corresponding to 96 samples are used as input. FASTQ reads are then aligned with the reference sequence using Smith Watermann affine score alignment strategy. Reads with a score below the predefined quality threshold are filtered out. This step drops all unwanted indels or mutations outside of the region of interest. Once the consensus is found, the reads are classified and quantified (number of reads, HDR, NHEJ, bp indels, etc).

The local alignment combined with block consensus ensures the best alignment score and it is particularly useful for ONT noisy data. Then, aligned sequence are classified into different categories based on the length of the indel. A predefined indel threshold drops out minor alignment groups (number below the defined threshold). The successfully aligned tracks are stored and indexed by unique aligned sequences. The webserver is written in Javascript, the data import and processing are performed at the client side. All output plots are exported into pie charts with separate alignment tracks rendered by HTML5 Canvas and Inline SVG. The entire program workflow is described in Figure 1.

### Data evaluation

Data evaluation by CRISRnano was performed using a Lenovo E590 (2.3 GHz Core i5, 8GB RAM) on Chrome version 99.0.4844.51. CRISPRnano also runs on current versions of Safari and Firefox.

## RESULTS

CRISPRnano is a web-based computational tool to qualitatively and quantitatively evaluate the outcomes of genome editing experiments. The user enters the reference locus sequence that matches the PCR amplicon, the predicted nuclease target site, and the gene name (optional). Raw sequencing data reads are loaded in FASTQ format, with up to 96 individual sequencing files analyzed in parallel. Our main goal was to develop a webserver that would allow scientists to screen genetically modified clones using inexpensive sequencers such as the MinION from ONT. One of the significant challenges we faced while building CRISPRnano was the low ONT read quality that is prone to false indels. Usually, these reads do not work with other webserver software because the alignment of the raw reads to the reference sequence does not allow gaps, especially close to the amplicon's 5′ and 3′. Also, ONT reads are usually long with variable read length. Therefore a global alignment approach using the Needleman Wunsch algorithm works poorly. To overcome these issues, CRISPRnano user interface allows the ability to limit the analysis to the predicted nuclease target site thus removing unintended gaps far away from the cleavage site. Moreover, the algorithm was developed by using adaptive Smith Watermann local alignment with affine gap penalty score that mitigates Oxford Nanopore sequencing errors. Using this approach CRISPRnano displays excellent output when analyzing noisy data and when compared to other publicly available software.

**Figure 2.**
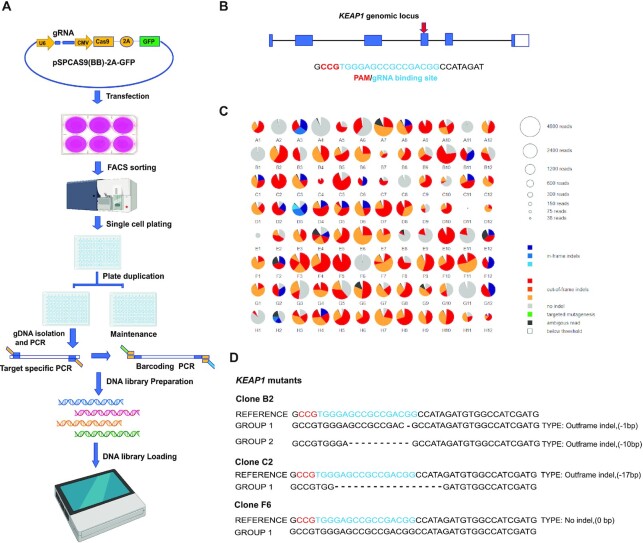
CRISPRnano experimental workflow. (**A**) Shown is the schematic view of the genome editing pipeline, from cell transfection to FASTQ data generation; (**B**) The genomic locus of the human *KEAP1* gene is depicted. Blue square represents coding exons. The red arrow highlights the target site of the CRISPR that is magnified below. (**C**) Example of an analysis performed by CRISPRnano on 96 clones. Every pie chart represents a clone, the size of each chart corresponds to the number of reads that were analyzed to evaluate the clone, and the color indicate the type of indel as described. (**D**), The identified indel mutations of three clones are depicted. Blue letters indicate the CRISPR target site, the indel size for each clone is depicted on the right.

CRISPRnano automatically proceeds with filtering low-quality reads, aligning reads to the reference amplicon, quantifying the proportion of knock-in events, and determining the proportion of frameshift (knockout) and in-frame mutations (Figure 1). In order to test the ability of CRISPRnano to detect indels and knock-in events, we used an established workflow (Figure 2A) to obtain gene-targeted cell clones for subsequent deep sequencing analysis. Experimental single-end deep sequencing Illumina data from 96 clones expressing Cas9, gRNA targeted to the *AHR* coding sequence, and a donor oligo were generated (supplementary Figure S1A). A graphical report is generated to visualize mutagenesis events (supplementary Figure S1B, C). Each pie chart contains all the important information, including the number of reads analyzed, alignment ratio, color-coded mutation types (in-frame, out-of-frame, knock-in), and a list of uniquely identified indel events that are interactively displayed. To further validate CRISPRnano ability to analyze low-quality deep sequencing reads using a MinION device (ONT), experimental Nanopore data from clones expressing Cas9, and a gRNA targeting *KEAP1* (Figure 2B) or *AHR* coding sequence were generated. CRISPRnano successfully identified *KEAP1* clones' genotype, as shown in Figure 2C, D. The same genotyping results were obtained when using Minimap2 and Samtools workflow (supplementary Figure S2) or an Illumina library (supplementary information). Furthermore, CRISPRnano correctly identified the genotype of *AHR* clones (supplementary information). Taken together, these data indicate that CRISPRnano can identify genetically modified clones using high (e.g. Illumina) or low quality reads (e.g. ONT).

Next, we wanted to determine the confidential number of reads necessary to identify a genotype due to the low quality of ONT reads. Thus, we validated our program by changing the number of available raw reads in a linear range from 200 to 10 000 reads randomly assigned in 8 replicas (supplementary Figure S3). While the number of successfully aligned reads is proportional to the number of raw reads, the indel percentage is almost identical in the 96 replicates (8 subsets). In our settings and when sequencing single diploid cell clones, we estimate that the number of reads per clone necessary to correctly identify mutants using ONT is around 1000 reads. However, with the recent improvement of nanopore basecaller, the ONT quality score can achieve a Phred Q + 20 equivalent to 0.99 accuracy. Therefore, a threshold of 0.05 equivalent to 500 reads per clone should allow a robust genotyping call. Illumina datasets feature an average quality score higher than Nanopore, thus a few hundred successfully aligned reads should be sufficient for genotyping purposes (13). By contrast, sequencing bulk of cells will require a higher number of reads (14), in our setup ∼2000 reads, and the sequencing coverage level will determine whether low frequency indel discovery can be made with a certain degree of confidence.

In our setup, using a minION sequencer with a Flongle flow cell and through multiplexing, it is possible to screen thousands of cell clones with an average per-clone sequencing cost of around 8 cents per clone.

## DISCUSSION

In summary, we developed CRISPRnano with an intuitive graphical user interface to help scientists identify genetically modified clones and general genotyping using portable and affordable ONT sequencers. CRISPRnano is a fast and reliable webserver evaluation tool for deep sequencing data analysis. The genotype analysis algorithm implemented in CRISPRnano can process reads (high or low fidelity) generated by virtually any type of sequencer. Our webserver works offline, it can adaptively handle long and noisy reads by empowering a more robust alignment algorithm, adaptive selection strategy, and concrete segment consensus. CRISPRnano performance is comparable with stand-alone aligner tools including Minimap2, Samtools, BCFtools that require manual curation efforts. Finally, user's data are not uploaded to the server, which is helpful to avoid risks associated with a GDPR violation (General Data Protection Regulation), security and privacy issues.

## DATA AVAILABILITY

The webserver can be accessed via the following link: www.crisprnano.de.

CRISPRnano is an open-source collaborative initiative available in the GitHub repository https://github.com/thachnguyen/CRISPRnano.

The sequence data generated for this study have been submitted to the NCBI Sequence Read Archive (SRA; http://www.ncbi.nlm.nih.gov/sra) under accession number PRJNA828591. CRISPRnano is available as open source software at http://www.CRISPRnano.de.

## Supplementary Material

gkac440_Supplemental_Files
