# Supplementary material for: Identification of genome edited cells using CRISPRnano
Source: Nucleic Acids Res. 2022 May 30;50(W1):W199–203. doi: 10.1093/nar/gkac440 (PMC9252781; doi:10.1093/nar/gkac440)

**Supplementary Information**

**
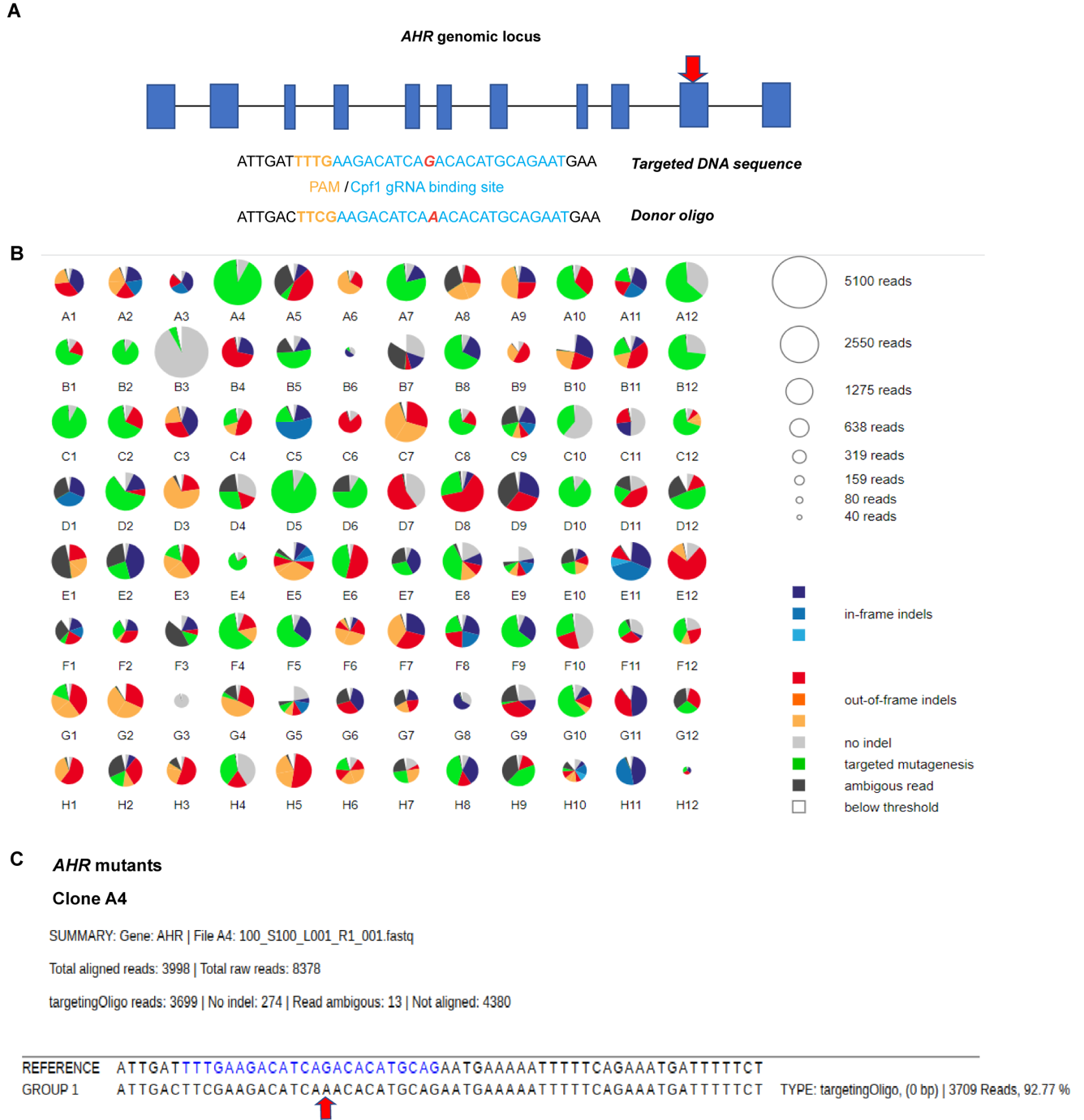
**

**Figure S1.** CRISPRnano analysis of Next Generation datasets (Illumina). A) AHR gene organization, reference, gRNA target and oligo sequences are depicted; B) Analysis performed by CRISPRnano of 96 AHR clones. Each pie chart represents a clone; C) A knock-in clone (A4) is depicted, arrow points to the nucleotide change (knock-in event).


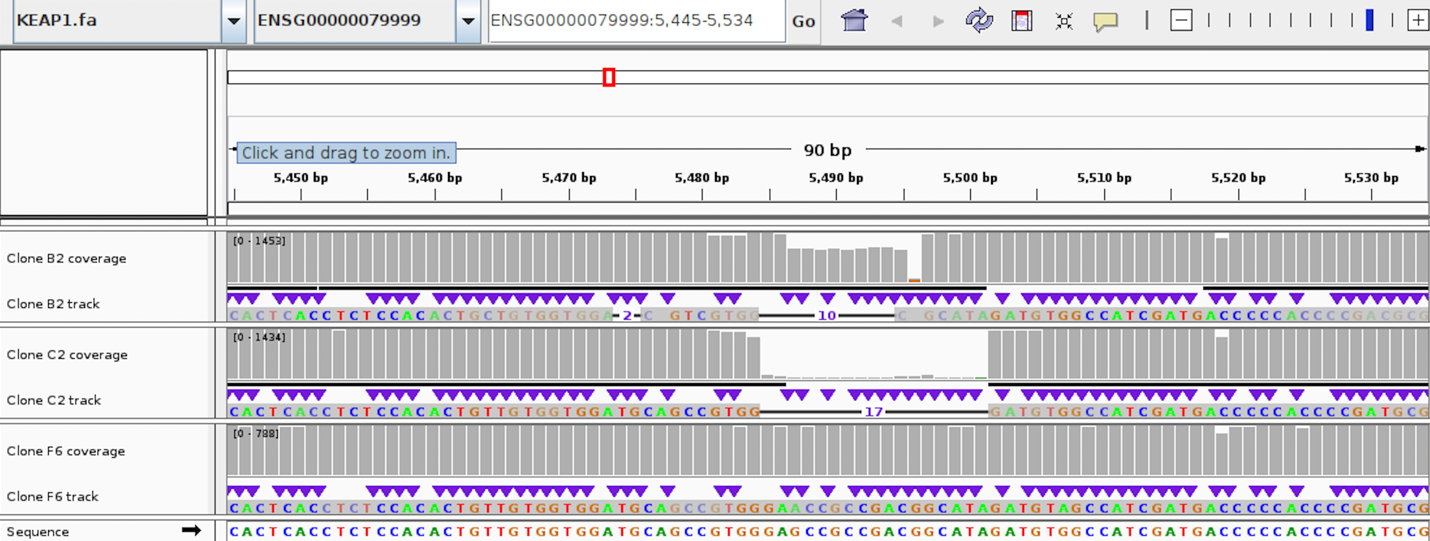


**Figure S2**. IGV alignment track using Minimap2 and Samtools workflow. Shown is the IGV schematic output of the KEAP1 clones alignment analysis using Minimap2 and SAM tool workflow. Depicted are three KEAP1 clones: heterozygous (top, deletion 1 bp and 10 bp, clone B2), homozygous (middle, deletion 17 bp, clone C2), and wildtype (bottom, clone F6)


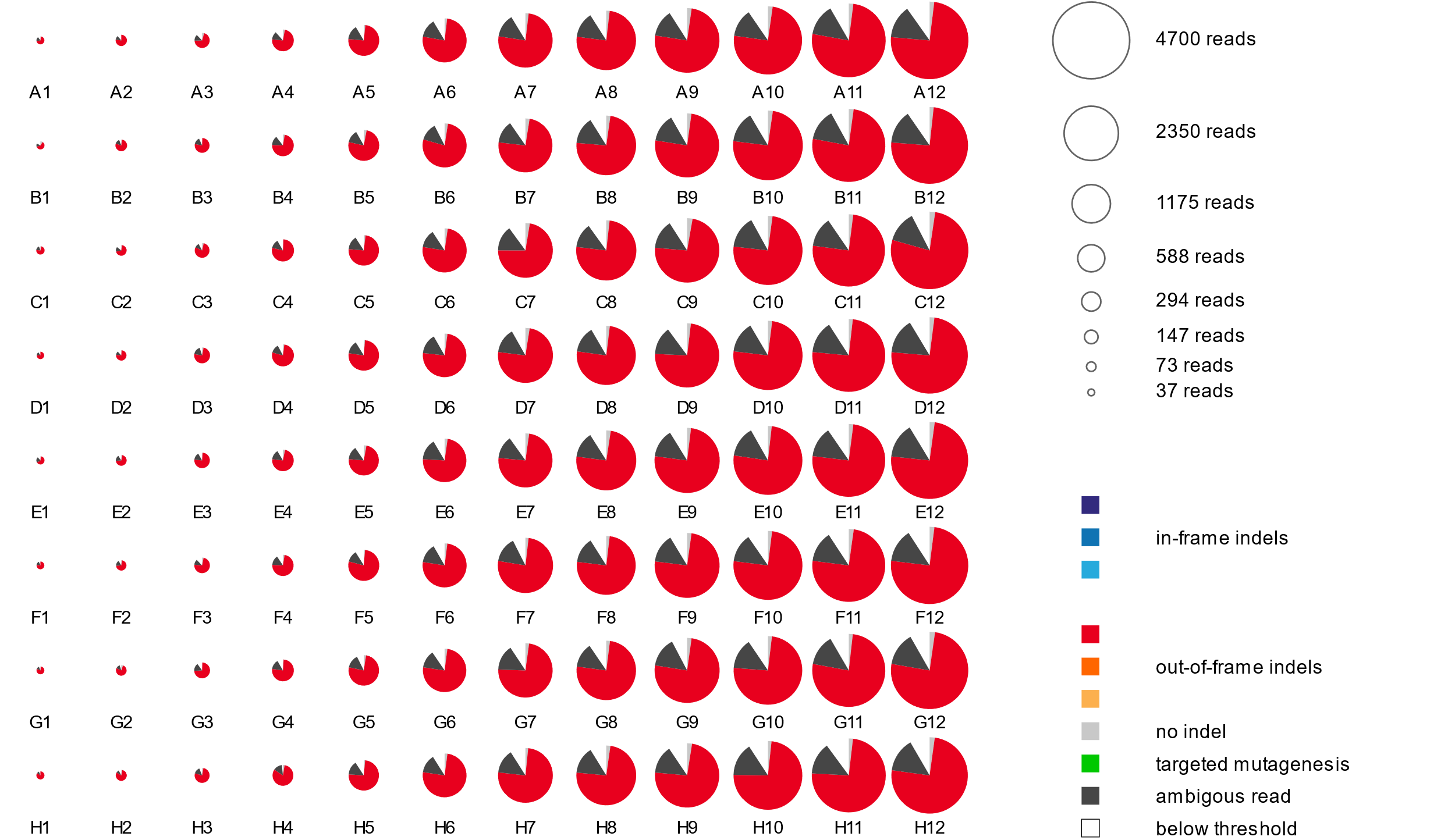


**Figure S3**. Validation of CRISPRnano using different read numbers and KEAP1 dataset. Each sample is randomly selected from 200 reads to 10000 read (linear) and separated into eight independent subsets.

**Comparison of CRISPResso2 and CRISPRnano analysis of ONT data**

CRISPResso2 and CRISPRnano were run in parallel to analyze two ONT datasets, *KEAP1* and *AHR*. CRISPResso2 can analyze up to 4 fastq files per run. In contrast, CRISPRnano can analyze up to 96 files at the client site, which leads to a faster analysis. Both software were compared by analysing one sample at the time.

***KEAP1***

Three samples (Fig. 2) were uploaded and evaluated using CRISPResso2 and CRISPRnano. CRISPResso2 analysis lasted for 40 seconds and successfully aligned <1% of the total reads. CRISPRnano successfully aligned the majority of the reads (average >50%) in less than 2 seconds.

***AHR***

Two samples were uploaded and evaluated using CRISPResso2 and CRISPRnano. Both CRISPResso2 and CRISPRnano worked succesfully. The performance of both and alignment are shown in the table below. For every single sample, CRISPRnano 's runtime was around three times faster than CRISPResso2.

|  | **CRISPRnano** | **CRISPResso2 (v2.1.3)** |
| --- | --- | --- |
| **Runtime (s)** | | |
| AHR_barcode01 | 122 | 381 |
| AHR_barcode02 | 130 | 442 |
| **Alignment statistic AHR_barcode01** | | |
| Number of raw read | 69085 | 69085 |
| Group1 | (-38 bp) \| 5935 Reads | (-38 bp) \| 3617 Reads |
| Group2 | (-29 bp) \| 5199 Reads | (-29 bp) \| 5250 Reads |
| Group3 | (-1 bp) \| 4017 Reads | (-1 bp) \| 4454 Reads |
| Group4 | (-1 bp) \| 1730 Reads | (-1 bp) \| 1960 Reads |
| **Alignment statistic AHR_barcode02** | | |
| Number of raw read | 77800 | 77800 |
| Group1 | (-19 bp) \| 4389 Reads | (-19 bp) \| 4735 Reads |
| Group2 | (-11 bp) \| 3044 Reads | (-11 bp) \| 3413 Reads |
| Group3 | (-1 bp) \| 2756 Reads | (-1 bp) \| 3111 Reads |
| Group4 | (-10 bp) \| 2657 Reads | (-10 bp) \| 3084 Reads |

**CRISPRnano analysis of Illumina and ONT data (comparison)**

CRISPRnano was validated using Illumina and ONT datasets. Each dataset contains 3 samples (ONT: KEAP1_wt, KEAP1_mt, KEAP1_hets, Illumina: KEAP1_29_S29_L001_R1_001_WT, KEAP1_61_S61_L001_R1_001_MT, KEAP1_40_S40_L001_R1_001_HETS). The result A1-A3 of each dataset has identical indexes.

The figures below show that CRISPRnano detected the same indels in a similar percentage using both, Illumina and ONT datsets. Alignment tracks are identical. Please note that the Illumina aligment track is reverse complemented.

ONT


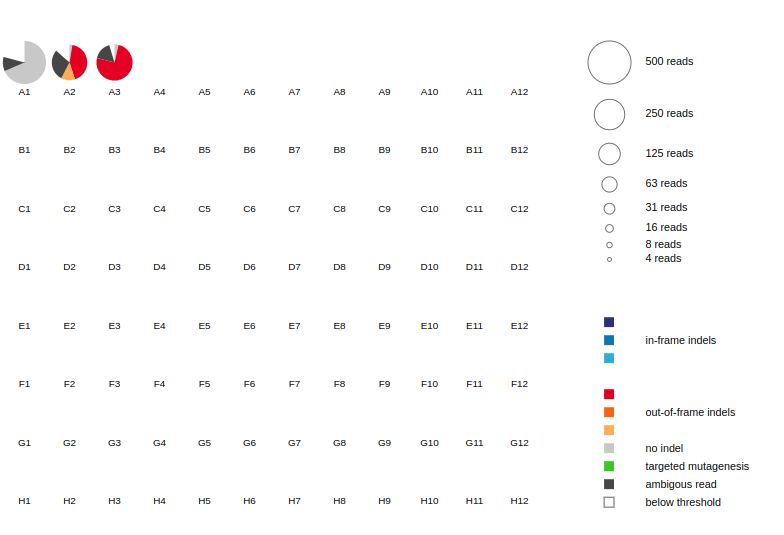


Illumina


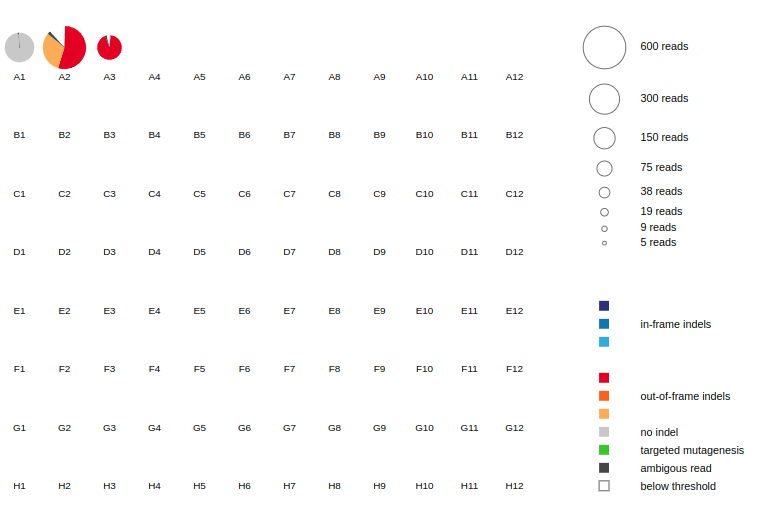


Well A1 (top: ONT, bottom: Illumina)


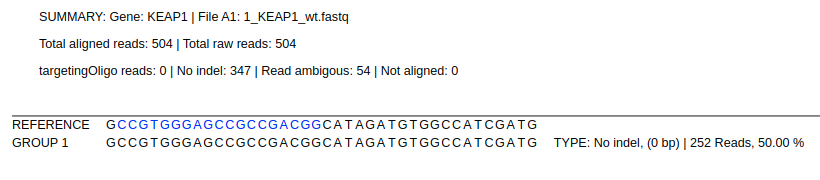


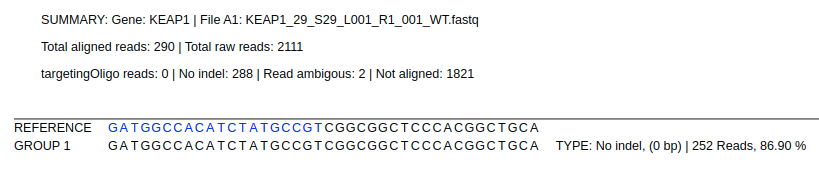


Well A2 (top: ONT, bottom: Illumina)


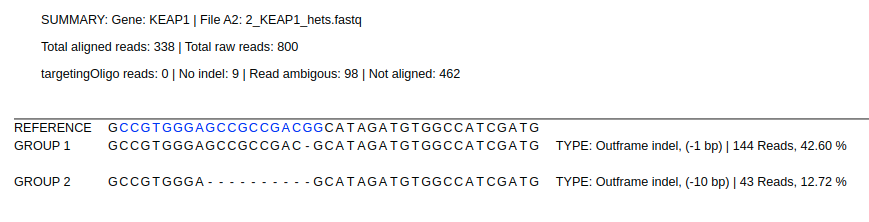


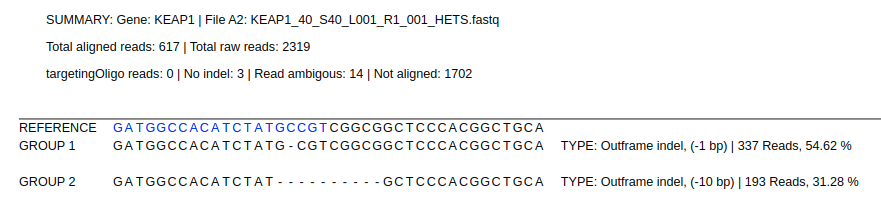


Well A3 (top: ONT, bottom: Illumina)


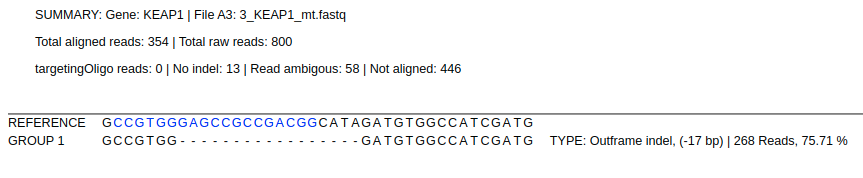


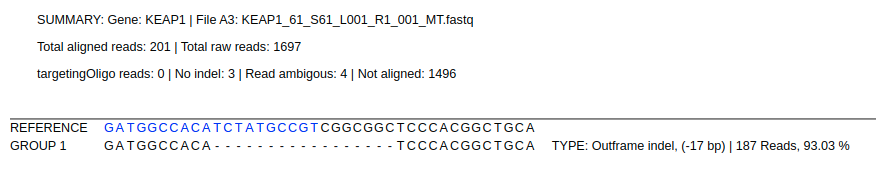


**Outknocker analysis of Illumina dataset (*KEAP1, same dataset as above*)**

Illumina datasets (*KEAP1*) were analyzed using Outknocker. Outknocker and CRISPRnano *KEAP1* genotyping is identical. Please note, ONT data are not aligned with Outknocker thus a comparison is not possible.


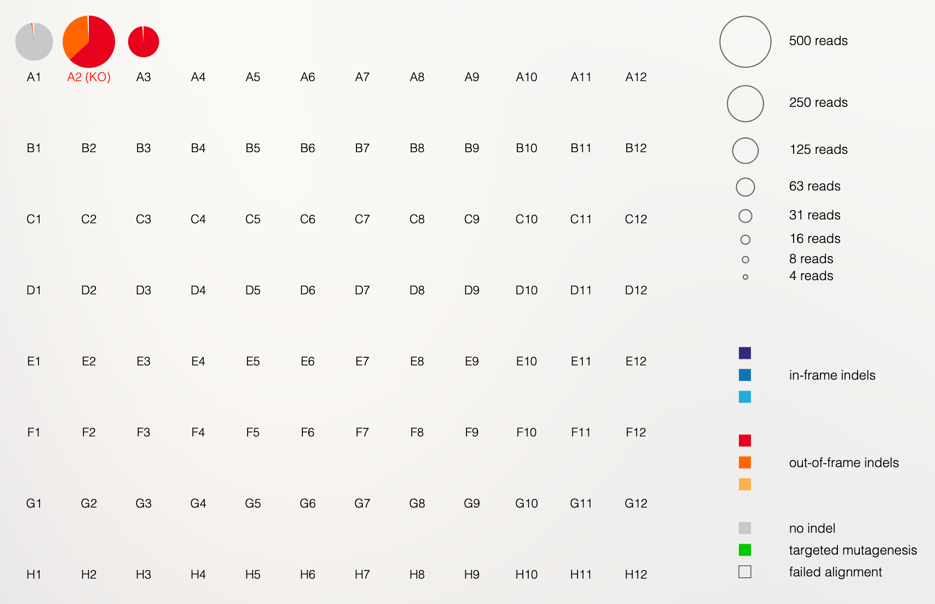


**
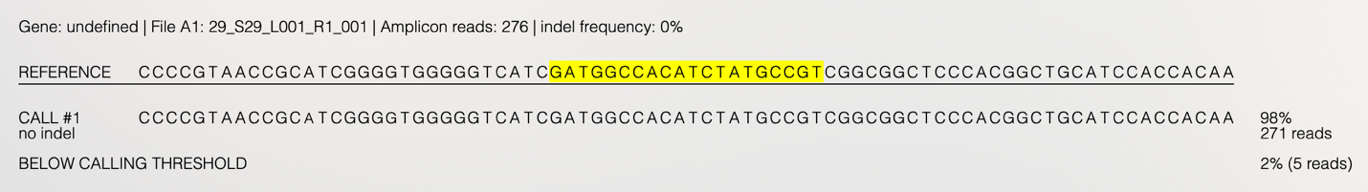
**

**
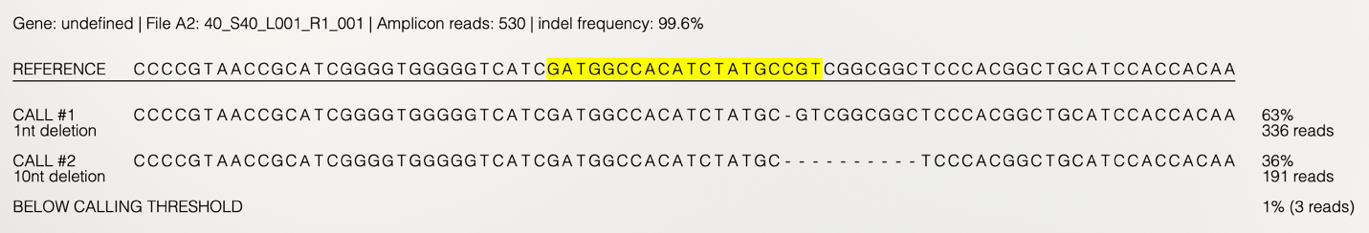
**

**
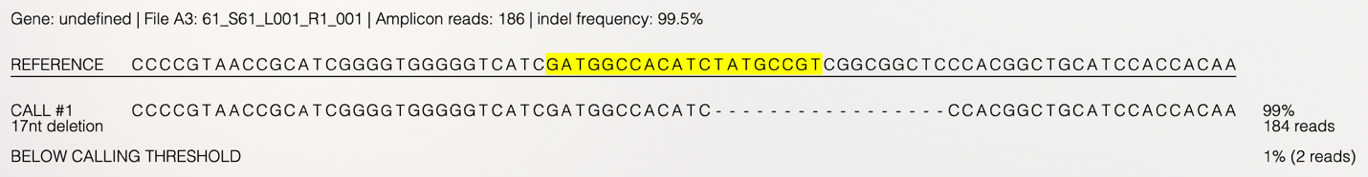
**

**Generation of in an silico test data script and comparison between CRISPRnano and Outknocker**

# Generate test data script

This python script generates two test files: short reads (300 nucleotides (NT) and long reads (~700 NT) which contain ratio of 30% WT, 20% 6 NT insertion, 30% 4 NT insertion and 20 % 5 NT deletion. We use these files to validate CRISPRnano and Outknocker

[1]: **from Bio import** SeqIO

*# Get reference gene, any gene, in this test we use HIF1A gene* ref_test = list(SeqIO.parse('/home/ag-rossi/ReferenceData/HIF1A.fa',␣ *,*_→_format='fasta'))[0]

**1.1 Generate long read test data.**

[2]: wt_long = ref_test[1100:1800] insert1 = ref_test[1100:1400]+'AATGGG'+ref_test[1400:1800] insert2 = ref_test[1100:1410]+'TTTA'+ref_test[1410:1800] del1 = ref_test[1100:1405]+ref_test[1410:1800]

fq_long_path ='long_test.fastq' **with** open(fq_long_path, "w") **as** fastq:

**for** i **in** range(300): record = wt_long record.letter_annotations["phred_quality"] = [40] * len(record)

SeqIO.write(record, fastq, "fastq") **for** i **in** range(200): record = insert1 record.letter_annotations["phred_quality"] = [40] * len(record)

SeqIO.write(record, fastq, "fastq") **for** i **in** range(300): record = insert2 record.letter_annotations["phred_quality"] = [40] * len(record)

SeqIO.write(record, fastq, "fastq") **for** i **in** range(200): record = del1 record.letter_annotations["phred_quality"] = [40] * len(record)

SeqIO.write(record, fastq, "fastq")

**1.2 Generate short read test data.**

[3]: wt = ref_test[1200:1500] insert1 = ref_test[1200:1400]+'AATGGG'+ref_test[1400:1500] insert2 = ref_test[1200:1410]+'TTTA'+ref_test[1410:1500] del1 = ref_test[1200:1405]+ref_test[1410:1500]

fq_long_path ='short_test.fastq' **with** open(fq_long_path, "w") **as** fastq:

**for** i **in** range(300):

record = wt record.letter_annotations["phred_quality"] = [40] * len(record)

SeqIO.write(record, fastq, "fastq") **for** i **in** range(200): record = insert1 record.letter_annotations["phred_quality"] = [40] * len(record)

SeqIO.write(record, fastq, "fastq") **for** i **in** range(300): record = insert2 record.letter_annotations["phred_quality"] = [40] * len(record)

SeqIO.write(record, fastq, "fastq") **for** i **in** range(200): record = del1 record.letter_annotations["phred_quality"] = [40] * len(record) SeqIO.write(record, fastq, "fastq")

We can use the reference and gRNA sequences below to test CRISPRnano and Outknocker

[4]:

*#Use this reference sequence*

print

(

'

Reference

**\n**

'

, wt_long

.

seq[

100

:

500

])

Reference

GATACTGAAAAAAGTATATGATGAGTGAATGAAATGCGGCACTAAAATGTTGCAAAAATTTTCGAACTCTGTCTCATTTTCCTGAAATTGAAGTATATTAAAGGAAAACCGTCAACATATATCTAAAGTAAGTAATCACTCGGTTAGAACTTAATGCAAGTTTTATAAATCACCTTGAAGTTTGAGTCTAAGGGGTACATTAGAGATTAAGAATTGTGAGTTGGACCAGTGGTGTTAAGAGCGGACTCCCCCATCCCCCAACACACACACAATTTTGCCCACTTTGGCATTTTAACTTTTAAGGAAATCACTTAAGGAATTGAAGATTTAGAGTAAGAGTTTTGGTTAGTAGACTGGCTTTGCTGTTAAATCCTTCCACTCTTCTGGCAGAGAGATTAAT

[5]:

*#gRNA*

print

(

'

gRNA

**\n**

'

, ref_test

.

seq[

1400

:

1420

])

gRNA TAGAGATTAAGAATTGTGAG

Our results show that the alignment tracks, the indel size and ratio are correct. Outknocker works when using short read test data but not with longer in silico amplicons.

**CRISPRnano output:**


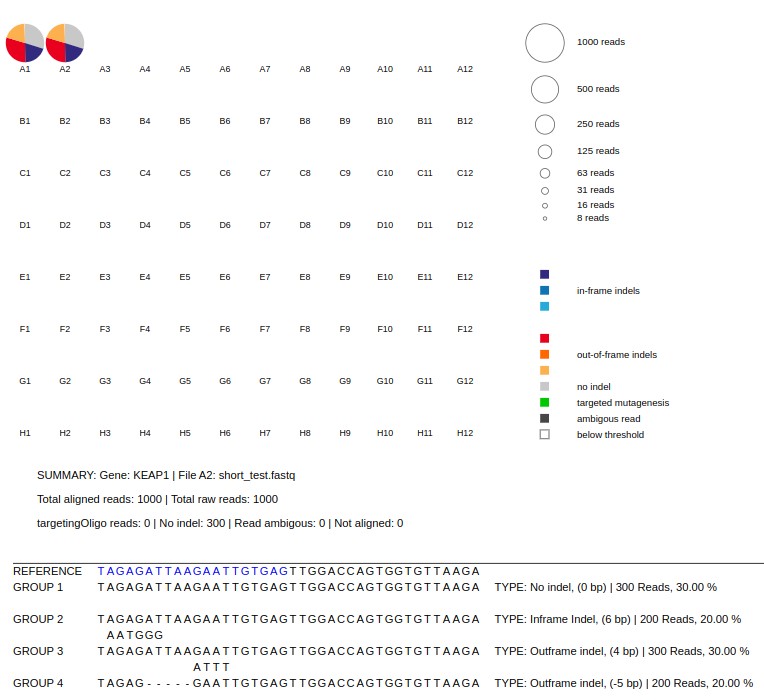


**Outknocker output:**


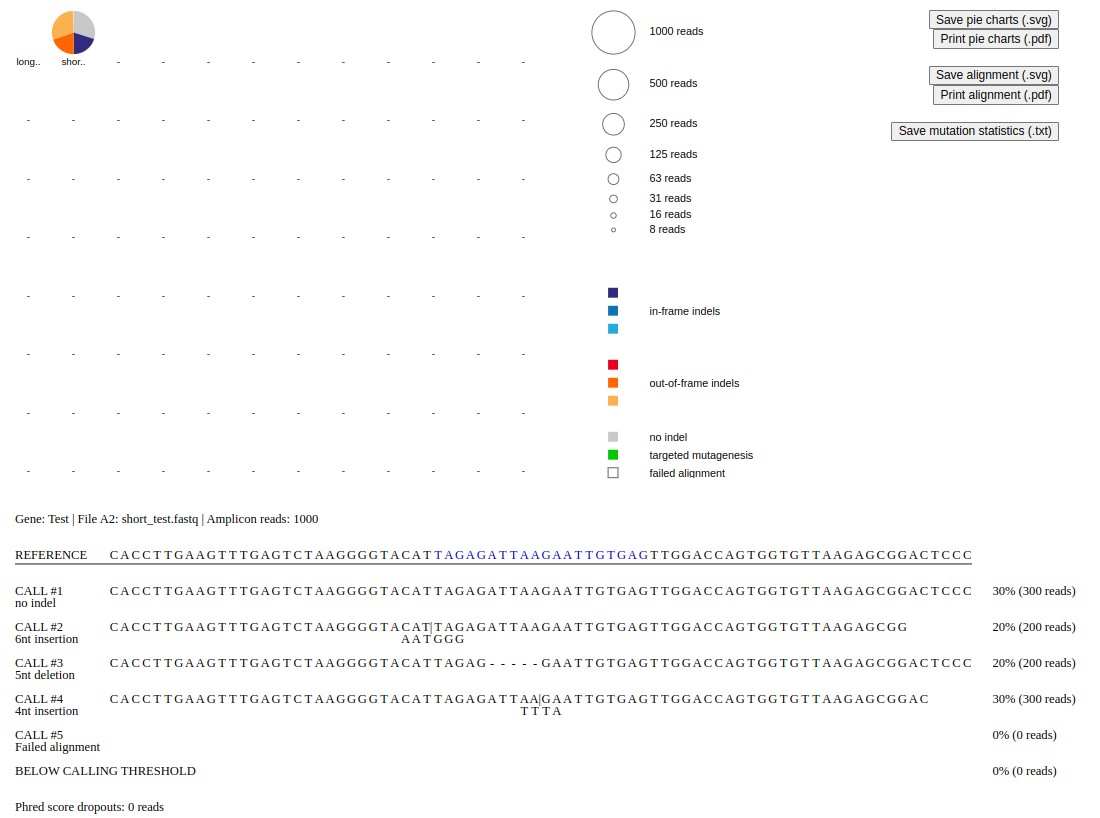


**CRISPR analyse workflow using Minimap2 and SAM tool**

**1 CRISPR validation script from raw data workflow**

dataset *KEAP1* Nanopore

# 1.1 Step1: Run basecaller

We recommend to rerun basecaller using sup model to improve ONT quality score.

[2]: !guppy_basecaller --min_qscore 7 -i fast5_pass/ -s fastq/ -c dna_r9.4. *,*_→_1_450bps_sup.cfg -x auto

ONT Guppy basecalling software version 6.1.2+e0556ff93, Minimap2 version

2.22-r1101

| config file: | /opt/ont/guppy/data/dna_r9.4.1_450bps_sup.cfg |
| --- | --- |
| model file: | /opt/ont/guppy/data/template_r9.4.1_450bps_sup.jsn |
| input path: | fast5_pass/ |
| save path: | fastq/ |
| chunk size: | 2000 |

chunks per runner: 208 minimum qscore: 7 records per file: 4000 num basecallers: 4

gpu device: auto kernel path: runners per device: 12

Use of this software is permitted solely under the terms of the end user license agreement (EULA).By running, copying or accessing this software, you are demonstrating your acceptance of the EULA. The EULA may be found in /opt/ont/guppy/bin Found 519 fast5 files to process. Init time: 1421 ms

0% 10 20 30 40 50 60 70 80 90 100%

|----|----|----|----|----|----|----|----|----|----|

***************************************************

Caller time: 1177954 ms, Samples called: 3397214922, samples/s: 2.884e+06 Finishing up any open output files. Basecalling completed successfully.

# 1.2 Step 2 (optional): Demultiplexed use ONT barcoder or customized barcoder

Barcode demultiplexing can be done by using our short script below, in this case our barcode is barcode_fw.fasta. Please note, it works only with Guppy_barcoder version 5.0.16.

[3]: **import os from Bio import** SeqIO **def** barcode_demux(fastq_path, result = 'barcoder', barcode_fw = 'barcode.

*,*_→_fasta'):

*'''Demultiplexing fastq by barcode*

*'''*

guppy_barcode_path= '/home/ag-rossi/projects/customized_barcode/data/' barcode_fasta = list(SeqIO.parse(barcode_fw, format='fasta')) f1 = open(guppy_barcode_path+'barcoding/barcode_arrs_cust2.cfg', 'r').

*,*_→_readlines() f1[-5] = 'last_index = **%i\n**'%**len**(barcode_fasta) f2 = open(guppy_barcode_path+'barcoding/barcode_arrs_cust2.cfg', 'w') f2.writelines(f1) f2.close()

**for** i,s1 **in** enumerate(barcode_fasta):

barcode_fasta[i].description = 'CUST**%02d**'%(i+1) barcode_fasta[i].name = 'CUST**%02d**'%(i+1) barcode_fasta[i].id = 'CUST**%02d**'%(i+1)

**with** open('/home/ag-rossi/projects/customized_barcode/data/barcoding/ *,*_→_custom_barcodes.fasta', "w") **as** output_handle:

SeqIO.write(barcode_fasta, output_handle, "fasta")

os.system('guppy_barcoder --input_path **%s** --save_path **%s** --data_path␣

*,*_→_**%s**barcoding --barcode_kits MY-CUSTOM-BARCODES --trim_barcodes -x␣

*,*_→_auto'%(fastq_path, result, guppy_barcode_path)) **return**

barcode_demux('fastq/pass', barcode_fw = 'barcode_rv.fasta')

**Then concatenate barcode files in each subfolder into one.**

# 1.3 Step 3: Aligned to reference genome

Minimap2 (version 2.17-r941) is used to align FASTQ sequences to the reference genome.

[44]: !minimap2 -t 16 -ax map-ont KEAP1.fa KEAP1/rep1_wt.fastq| samtools view -Sb |␣ *,*_→_samtools sort - -o BAMs/rep0.bam

!minimap2 -t 16 -ax map-ont KEAP1.fa KEAP1/rep2_hets.fastq| samtools view -Sb␣ *,*_→_| samtools sort - -o BAMs/rep1.bam

!minimap2 -t 16 -ax map-ont KEAP1.fa KEAP1/rep3_mt.fastq| samtools view -Sb |␣ *,*_→_samtools sort - -o BAMs/rep2.bam

[M::mm_idx_gen::0.001*1.97] collected minimizers

[M::mm_idx_gen::0.003*5.41] sorted minimizers

[M::main::0.003*5.37] loaded/built the index for 1 target sequence(s)

[M::mm_mapopt_update::0.003*4.92] mid_occ = 23

[M::mm_idx_stat] kmer size: 15; skip: 10; is_hpc: 0; #seq: 1

[M::mm_idx_stat::0.003*4.69] distinct minimizers: 2869 (92.82% are singletons); average occurrences: 1.167; average spacing: 5.205 [M::worker_pipeline::0.035*9.28] mapped 504 sequences

[M::main] Version: 2.17-r941

[M::main] CMD: minimap2 -t 16 -ax map-ont KEAP1.fa KEAP1/rep1_wt.fastq

[M::main] Real time: 0.036 sec; CPU: 0.329 sec; Peak RSS: 0.035 GB

[M::mm_idx_gen::0.001*1.88] collected minimizers

[M::mm_idx_gen::0.003*5.81] sorted minimizers

[M::main::0.003*5.78] loaded/built the index for 1 target sequence(s)

[M::mm_mapopt_update::0.003*5.21] mid_occ = 23

[M::mm_idx_stat] kmer size: 15; skip: 10; is_hpc: 0; #seq: 1

[M::mm_idx_stat::0.003*4.97] distinct minimizers: 2869 (92.82% are singletons); average occurrences: 1.167; average spacing: 5.205 [M::worker_pipeline::0.067*10.44] mapped 800 sequences

[M::main] Version: 2.17-r941

[M::main] CMD: minimap2 -t 16 -ax map-ont KEAP1.fa KEAP1/rep2_hets.fastq

[M::main] Real time: 0.068 sec; CPU: 0.700 sec; Peak RSS: 0.041 GB

[M::mm_idx_gen::0.001*2.21] collected minimizers

[M::mm_idx_gen::0.002*6.67] sorted minimizers

[M::main::0.002*6.62] loaded/built the index for 1 target sequence(s)

[M::mm_mapopt_update::0.002*5.91] mid_occ = 23

[M::mm_idx_stat] kmer size: 15; skip: 10; is_hpc: 0; #seq: 1

[M::mm_idx_stat::0.003*5.57] distinct minimizers: 2869 (92.82% are singletons); average occurrences: 1.167; average spacing: 5.205 [M::worker_pipeline::0.065*10.43] mapped 800 sequences

[M::main] Version: 2.17-r941

[M::main] CMD: minimap2 -t 16 -ax map-ont KEAP1.fa KEAP1/rep3_mt.fastq

[M::main] Real time: 0.067 sec; CPU: 0.682 sec; Peak RSS: 0.043 GB

[45]:

!

samtools index BAMs/rep0.bam

!

samtools index BAMs/rep1.bam

!

samtools index BAMs/rep2.bam

## 1.3.1 Get sgRNA location

[46]: **from Bio import** SeqIO reference = list(SeqIO.parse('KEAP1.fa', format='fasta'))[0] sgRNA = 'CCGTGGGAGCCGCCGACGG' print('Location of sgRNA **%i**'%**reference**.seq.index(sgRNA))

Location of sgRNA 5478

Then we can use IGV to view each alignment track at the desired locus to validate the indel position.

## 1.3.2 Step 4: bcftools to create VCF file

We can see VCF files or IGV coverage track to detect the indel region.

[47]: !bcftools mpileup --fasta-ref KEAP1.fa BAMs/rep0.bam> file0.mpileup

!bcftools view file0.mpileup > rep0.vcf

!bcftools mpileup --fasta-ref KEAP1.fa BAMs/rep1.bam> file1.mpileup

!bcftools view file1.mpileup > rep1.vcf

!bcftools mpileup --fasta-ref KEAP1.fa BAMs/rep2.bam> file2.mpileup

!bcftools view file2.mpileup > rep2.vcf

[mpileup] 1 samples in 1 input files

[mpileup] maximum number of reads per input file set to -d 250

[mpileup] 1 samples in 1 input files

[mpileup] maximum number of reads per input file set to -d 250

[mpileup] 1 samples in 1 input files

[mpileup] maximum number of reads per input file set to -d 250

[48]:

**import**

**igv**

b

=

igv

.

Browser({

"

genome

"

:

"

hg38

"

})

b

.

locus

b

.

load_track(

{

"

name

"

:

"

WT

"

,

"

url

"

:

"

BAMs/rep0.bam

"

,

"

format

"

:

"

bam

"

,

"

indexed

"

:

**True**

})

b

.

load_track(

{

"

name

"

:

"

HETS

"

,

"

url

"

:

"

BAMs/rep1.bam

"

,

"

format

"

:

"

bam

"

,

"

indexed

"

:

**True**

})

b

.

load_track(

{

"

name

"

:

"

MT

"

,

"

url

"

:

"

BAMs/rep2.bam

"

,

"

format

"

:

"

bam

"

,

"

indexed

"

:

**True**

})

b

.

show()

<

IPython.core.display.HTML object

>

**Smith Watermann alignment strategy**:

CRISPRnano use Smith Watermann with affine score alignment. Algorithm and formulas are from (Durbin et al., 1998; Smith & Waterman, 1981).

Let *X = x_1_ x_2_ ...x_n_, Y = y_1_ y_2_...y_m_* are two sequences we align. In this case *X* is our read and *Y* is the reference genome.

Step1: Determine the substitution matrix S: $s\left( x_{i},y_{i} \right)$ for each j, j and affine gap penalty

$$\gamma\left( g \right)=-d -\left( g-1 \right)e$$

Where *g* is gap’s length, *d* is gap open penalty, *e* is gap extent penalty.

Step 2: Construct a scoring matrix *F* and assign zero to the first row and column.

Step 3: For each position i, j we assign new value of matrix F:

$F\left( i,j \right)=max\left\{ \begin{matrix} F(i-1, j-1) + s\left( x_{i},y_{i} \right) \\ I_{x}\left( ⅈ-1, j-1 \right) + s\left( x_{i},y_{i} \right) \\ I_{y}\left( ⅈ-1, j-1 \right)+ s\left( x_{i},y_{i} \right) \end{matrix} \right.$

In this formula, $F\left( i,j \right)$ is the best score up to position $\left( x_{i},y_{i} \right)$. $I_{x}\left( ⅈ, j \right)$ and $I_{y}\left( ⅈ, j \right)$ are the best score *x_i_,* and *y_j_* align to the gap (*d*: gap open or *e:* gap extend).

$$I_{x}\left( ⅈ, j \right)=max\left\{ \begin{matrix} F\left( i-1, j \right) - d \\ I_{x}\left( ⅈ-1, j \right) - e \end{matrix} \right.$$

$$I_{y}\left( ⅈ, j \right)=max\left\{ \begin{matrix} F\left( i, j-1 \right)-d \\ I_{x}\left( ⅈ, j-1 \right) - e \end{matrix} \right.$$

Step 4: Alignment route traceback: we start from *F_max_* and end at 0, the route generates our local alignment.

**Genotyping bulk of cells using CRISPRnano and Outknocker**

An Illumina dataset (*AHR*) was analyzed using Outknocker and CRISPRnano. Outknocker and CRISPRnano genotyping outputs are comparable.

**CRISPRnano output:**


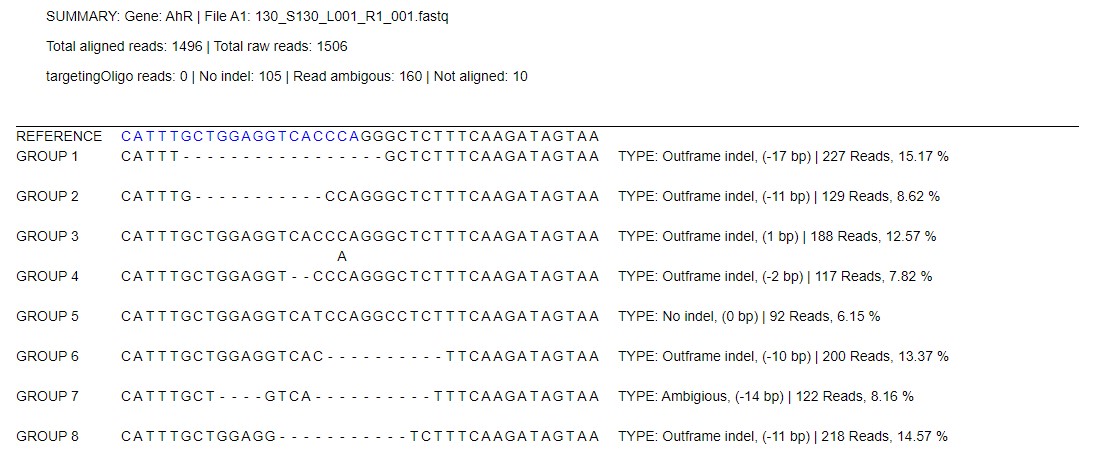


**Outknocker output:**


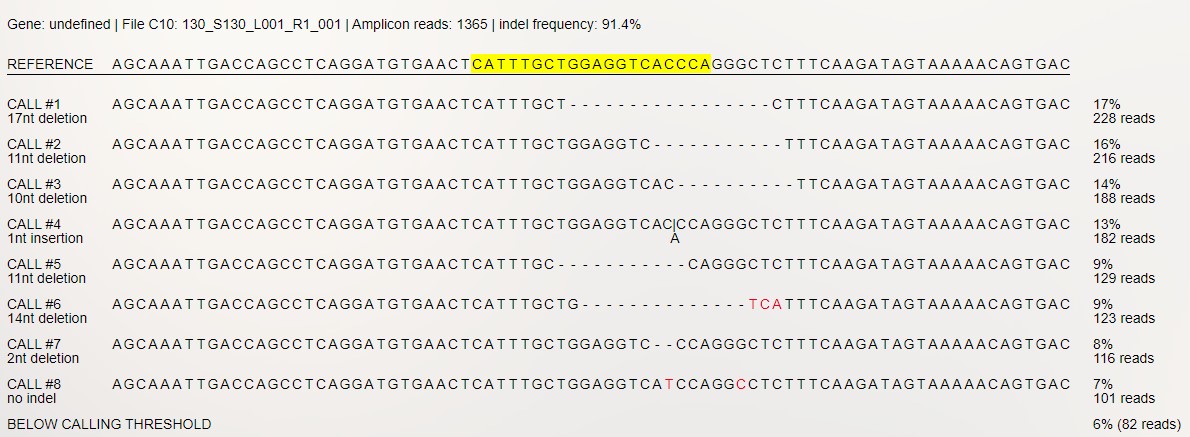

Supplement: gkac440_Supplemental_Files [file gkac440_supplemental_files.zip › Supplementary information_Rev2.docx]
